# Development of core competency indicators for emergency medicine residents based on the competency model: a Delphi and analytic hierarchy process study

**DOI:** 10.1186/s12909-026-09876-3

**Published:** 2026-07-07

**Authors:** Caixia Yu, Wanwei Guo, Linna Yu, Chongming Huang, Guoxiang Zhao

**Affiliations:** 1 Department of Science and Education, The Affiliated Guangdong Second Provincial General Hospital of Jinan University, Guangzhou, 510310 P. R. China; 2 Emergency Department, The Affiliated Guangdong Second Provincial General Hospital of Jinan University, Guangzhou, 510310 P. R. China

**Keywords:** Emergency medicine, Standardized residency training, Competency model, Delphi method, Core competency

## Abstract

**Background:**

Standardized residency training in emergency medicine is not well-defined in China. Establishing a core competency indicator system for emergency residency training is essential for standardizing and enhancing the quality of training and emergency care.

**Objectives:**

This study aimed to develop a core competency indicator system for emergency residency training in China, based on the competency iceberg model.

**Methods:**

This study employed a multi-step approach, including a literature review, semi-structured expert interviews, to formulate a list of preliminary indicators. A two-round modified Delphi method was utilized to achieve consensus, with 19 emergency experts recruited from 11 tertiary hospitals across four regions of China. Consensus was determined using a 5-point Likert scale, with a mean importance score > 3.5, a coefficient of variation < 0.20, and a full-score proportion > 20% serving as criteria for indicator retention.

**Results:**

All 19 invited experts completed both rounds of consultation, achieving a 100% valid response rate. The authority coefficient of the consultation was 0.895, and Kendall’s W values ranged from 0.566 to 0.634 (*p* < 0.005), indicating strong expert consensus. The final indicator system includes 34 third-level indicators within 14 s-level indicators, categorized into 5 first-level dimensions: motivation, trait, self-concept, knowledge, and skill.

**Conclusions:**

This study represents an initial step towards establishing a contemporary competency-based training program for emergency residency training in China, providing a scientific reference for the standardization of emergency residency training.

**Supplementary Information:**

The online version contains supplementary material available at https://doi.org/10.1186/s12909-026-09876-3.

## Introduction

The emergency department (ED) stands as the critical frontline in hospitals for managing acute and life-threatening conditions, where the quality and efficiency of care are directly tied to patient survival and clinical outcomes [1]. In China, the standardized residency training program represents a pivotal phase for developing and shaping the clinical competencies of future emergency medicine practitioners. Nevertheless, the current competency cultivation system for emergency medicine residents in China lacks a unified, scientific, and systematic framework of core competency standards to guide its implementation [2]. The absence of such a standard can lead to significant variability in the proficiency levels of graduating physicians. This variability may, in turn, contribute to clinical misjudgments, delays in treatment, and other adverse events. These issues not only heighten risks within the ED workflow but also pose a latent threat to overall healthcare quality and patient safety [3]. Consequently, constructing a core competency indicator system that aligns with the distinctive realities of emergency medicine practice has emerged as a crucial and pressing task to enhance training quality and safeguard emergency care.

Against this backdrop, this study aimed to address two core research questions by introducing the competency iceberg model:

1. What are the core competencies required for Chinese emergency medicine residents after standardized training, covering both explicit clinical abilities and implicit professional traits?
2. How to develop a scientific, systematic and quantifiable core competency indicator system tailored to the context of Chinese emergency practice?

Scholars globally have undertaken valuable explorations in constructing competency models within medical education. Internationally, frameworks such as the Canadian CanMEDS Physician Competency Framework and the core competency model from the American Accreditation Council for Graduate Medical Education (ACGME) have gained widespread adoption. These models consistently emphasize multidimensional abilities encompassing medical knowledge, clinical skills, and communication and collaboration [4]. Domestically, some research has introduced competency models into medical education, proposing preliminary competency requirements specific to the emergency department [5]. A common limitation, however, is that many of these studies concentrate on isolated dimensions, such as resuscitation skills, failing to achieve a systematic integration of knowledge, skills, attitudes, and professional behaviors essential for holistic practice [6].While existing research has identified key abilities required of emergency physicians—including rapid patient assessment, management of critical illnesses, and multi-disciplinary collaboration [7]—a cohesive, hierarchy-clear, and behaviorally-anchored set of core competency indicators grounded in a competency model remains to be established.

A comprehensive review reveals primary shortcomings in three aspects. There is a notable lack of systemic integration; most studies focus narrowly on a specific category of ability, like technical procedures, without synthesizing the diverse elements required for competent emergency practice into a unified model [8]. Furthermore, contextual applicability is limited. Mature physician competency models developed internationally, such as CanMEDS and the ACGME competencies, originate from specific healthcare and educational ecosystems. Their competency domains and assigned weights often do not adequately incorporate contextual factors prevalent in Chinese EDs, including exceptionally high patient volumes (the annual patient volume of a single tertiary hospital ED in China can reach 150,000–300,000, far outpacing the average ED volume in Western healthcare systems), prolonged waiting times (the average waiting time for emergency patients in large urban medical centers can exceed 6 h during peak operational periods), severe departmental overcrowding, high rates of unscheduled 72-hour return visits (approximately 15% of emergency patients return to the ED within this timeframe), as well as distinct challenges in clinical safety management under high-workload conditions [9]. These contextual factors create unique competency requirements for emergency residents in China, which are not fully reflected in international models. This discrepancy can lead to potential misalignment when these models inform the setting of localized training objectives and curriculum design. These limitations collectively contribute to ambiguous training goals for emergency medicine residents and unpredictable training outcomes. Over time, this situation may compromise the overall caliber of the specialist workforce in emergency medicine, ultimately constraining the enhancement of emergency care service quality.

To address these gaps, this study aims to develop a set of core competency indicators tailored to the clinical practice context of emergency medicine in China, based on a competency model. The research will employ a combined methodology of literature analysis, expert interviews, and the Delphi expert consultation technique to systematically screen, integrate, and validate potential indicators. Multiple rounds of expert consensus will be conducted to ensure the scientific rigor and practical utility of the final indicator system. The scope of this study is focused on resident trainees within the emergency departments of tertiary hospitals in China. The core competencies to be encompassed include multiple dimensions such as clinical practice, medical knowledge, professionalism, and communication and collaboration. This study does not extend to the training of specialist emergency physicians or to training conducted in primary care institutions.

## Methods

### Ethical considerations

This study adhered to the ethical principles outlined in the World Medical Association Declaration of Helsinki for Medical Research Involving Human Subjects. Detailed information was provided to the experts through email, elucidating the study’s objectives, methodology, confidentiality measures, and data management. This anonymous Delphi survey, classified as having negligible risk, ensured voluntary participation, with each expert providing informed consent through email by providing an affirmative reply. This study was approved by the ethics board of The Affiliated Guangdong Second Provincial General Hospital of Jinan University (Approval ID: 2026-KY-KZ-061-01).

### Design

This study utilized two rounds of Delphi survey based on a list of preliminary indicators and was conducted from March to May 2025. A five-member research panel was established by the research team, comprising individuals with expertise in emergency medicine, medical education, and health policy. Collaborative discussions involving the research team led to conclusive decisions when uncertainties regarding indicator inclusion arose.

### Generating preliminary indicators

This study is grounded in the competency model as its theoretical foundation. Originally proposed by McClelland in 1973, this model emphasizes the integrated manifestation of knowledge, skills, attitudes, and behaviors within a specific professional role [10]. It operates by defining explicit competency objectives, measurable performance criteria, and dynamic feedback mechanisms, all aimed at achieving predetermined standards of proficiency. In recent years, this framework has seen extensive application in enhancing diverse areas, including clinical practice, leadership, collaborative skills, and educational competencies [11].

Specifically, we adopted Spencer’s classic Iceberg Competency Model, which categorizes professional competencies into two categories: explicit ‘surface’ competencies (knowledge and skill) that are visible and trainable, and implicit ‘submerged’ competencies (self-concept, trait, and motivation) that are deep-seated and determine long-term performance. This framework guided us to develop the dimension of our initial indicator system for emergency medicine residents.

#### Literature review

A preliminary list of indicators was generated through a combination of literature review and semi-structured interviews. Firstly, a literature review was conducted using search terms as “competency”, “competency model”, “emergency medicine”, “residency training”, “standardized residency training”, “core competency indicators”, “Delphi method”. The search covered multiple databases including PubMed, CINAHL, ERIC, Wanfang Data, and China National Knowledge Infrastructure (CNKI) from January 2013 to December 2025. Initially, a total of 217 references were retrieved. Following title or abstract screening, non-relevant references were excluded, and the remaining 41 references underwent a thorough assessment of their full text for eligibility. Ultimately, 30 references were included.

Two researchers independently screened all the included references to extract potential indicators based on specific criteria: (1) relevance to emergency residency training, (2) pertinence to the training requirements, and (3) alignment with the training objectives.

#### Semi-structured interview

Purposive sampling was employed to invite 5 senior emergency specialists from tertiary hospitals for semi-structured interviews. All participants had over 10 years of experience in emergency clinical practice or residency training, to ensure their input could reflect the actual needs of emergency training in China. The interview guide was designed strictly based on the five dimensions of Spencer’s Iceberg Competency Model, covering both explicit ‘above-the-iceberg’ surface competencies (knowledge and skill) and implicit ‘submerged’ deep competencies (self-concept, trait, and motivation), to fully explore the core competencies required for emergency residents in the Chinese context. During the interviews, participants were asked to elaborate on the relevant questions based on the pre-designed interview guide (Table 1). All interviews were conducted face-to-face or via online meeting, with an average duration of 50 min. Interview data were processed through a systematic workflow including audio recording, verbatim transcription, and structured coding. For the subsequent thematic analysis, we adopted the standardized six-phase framework recommended by Braun & Clarke [12]:

1. Familiarization: Two independent researchers repeatedly read all interview transcripts to familiarize themselves with the full dataset.
2. Initial coding: The transcripts were then coded line-by-line using an inductive approach, to identify meaningful segments relevant to emergency resident competencies, without applying pre-defined theoretical categories.
3. Searching for themes: Coded segments were then collated into candidate themes, with similar codes grouped together based on semantic content.
4. Reviewing themes: These candidate themes were then reviewed against the raw interview data and the five dimensions of the iceberg competency model, to verify that the themes were internally coherent and aligned with the study’s research objectives.
5. Defining and naming themes: Each confirmed theme was then clearly operationalized and named, with specific competency indicators extracted from the coded data under each theme.
6. Report production: The final list of interview-derived indicators was compiled, and subsequently integrated with indicators identified from the literature review to generate the preliminary indicator pool.

To ensure inter-coder reliability, both independent researchers received standardized training on the thematic analysis procedure prior to coding, and were provided with a standardized codebook that outlined the study’s objectives and the operational definitions of competency indicators, to standardize their coding process. Any discrepancies between the two researchers’ coding results were resolved through consensus-based discussion with the full research panel, to ensure the reliability of the extracted themes and indicators. To illustrate the practical insights derived from these interviews, we present representative responses from the participating experts:

1. A senior emergency physician with 18 years of clinical experience noted: “In our tertiary ED, we receive over 250 patients daily during peak hours. For residents, the most critical ability is not just how to perform intubation, but how to triage 10 incoming patients at once and identify the critical ones within 2 minutes—if you miss that, the whole department will be backed up.”
2. Another expert in residency training emphasized: “New residents often focus too much on learning technical skills, but what really separates good emergency doctors is how they stay calm when 3 critical cases come in at the same time. That stress resilience can’t be taught in a textbook, it’s something we need to assess and train for from the start.”
3. A third specialist pointed out: “In China, our EDs have much higher patient volumes than Western hospitals. Residents need to be able to communicate with anxious patients and families quickly, otherwise small issues can turn into conflicts. Empathy isn’t just a nice-to-have, it’s essential to keep the department running smoothly.”

These qualitative insights enabled us to identify implicit competency indicators that were not fully captured in existing literature, such as stress resilience and rapid communication skills, which were subsequently validated through the Delphi consensus process.

**Table 1:** Semi-structured interview guide

Competency Dimension	Interview Questions
Knowledge (Explicit ‘above-the-iceberg’ competency)	1. What basic medical knowledge do you think a qualified emergency resident must master proficiently?
	2. In terms of clinical professional knowledge, which areas of knowledge are the most critical?
	3. Besides medical knowledge, what other related fields of knowledge are helpful for emergency work?
Skill (Explicit ‘above-the-iceberg’ competency)	1. Could you list some clinical operation skills that emergency residents must master proficiently?
	2. Among non-operative skills, which ones are crucial?
	3. How do you evaluate the current performance of residents in these skills? What are the common strengths or deficiencies?
Self-concept (Implicit ‘submerged’ competency)	1. In your opinion, what is the most important professional quality or spirit of an excellent emergency physician?
	2. How do you view your own role and position in the emergency team?
	3. When you encounter an uncertain diagnosis or treatment plan, what are your inner thoughts? What would you usually do?
	4. How do you view errors or omissions in clinical work? How do you think we should face and deal with them?
Trait (Implicit ‘submerged’ competency)	1. What characteristics of your personality are particularly suitable (or unsuitable) for emergency department work?
	2. Could you share an experience of successfully treating a patient under extreme pressure? What personal qualities do you think helped you get through this situation?
	3. After continuous high-intensity work, how do you adjust your state and emotions? Do you think this self-regulation ability is important?
Motivation (Implicit ‘submerged’ competency)	1. What initially made you choose the medical major, and then chose emergency medicine as your specialty?
	2. At work, what brings you the most sense of achievement and satisfaction?
	3. Facing the high risk, high intensity and sometimes misunderstood situations in work, what supports you to persist and keep moving forward?
	4. What are your expectations and goals for your future career development? How do you think emergency work experience can help you achieve these goals?

#### Delphi phase

A Delphi procedure was utilized to establish consensus on the indicator system. All potential experts were recruited from 11 tertiary hospitals across four regions of China (South China, North China, Southwest China, East China and Northeast China). The inclusion criteria for experts were: (1) more than 5 years of clinical or educational experience in emergency medicine; (2) active involvement in emergency residency training; (3) a professional title of senior level; (4) a bachelor’s degree or higher; (5) willingness to participate.

Experts completed the Delphi questionnaires using a text file attached to the invitation email. Each round allowed a two-week window for scoring, with reminder emails sent a week before the deadline to enhance response rate. To maintain objectivity, experts did not interact with each other and were not informed of the identities of their fellow experts. After each round, an anonymized summary of the combined results from the prior round was provided to the experts.

The degree of agreement regarding the importance of each indicator was rated using a 5-point Likert scale with the following options: “Not at all important”, “Slightly Important”, “Important”, “Fairly Important” and “Extremely Important”. In addition, experts were asked to rate their familiarity with each indicator and their judgment basis.

#### Data analysis

Data underwent dual-entry verification in Excel 2024 and SPSS 25.0 for statistical analysis. Descriptive analyses included mean importance scores, standard deviations (SD), and composition ratios. Indicator weights at all tiers were determined using Analytic Hierarchy Process (AHP) software, with consistency tests conducted to ensure the rationality of the weights. The reliability and validity of expert consultation results were validated through expert response rate, authority coefficient, and Kendall’s concordance coefficient.

## Results

### Expert demographic profile

A total of 19 emergency medicine experts were enrolled in this study, and all of them completed the two rounds of Delphi consultation, with a 100% response rate in both rounds. The detailed demographic characteristics of the experts are presented in Table 2. With regard to the professional field categorization of the participating specialists presented in Table 2, all participating experts were categorized under the broad umbrella category of “Clinical Medicine” within China’s professional title credentialing system. While emergency medicine has been recognized as an independent clinical specialty in practice since 2009, the formal specialty taxonomy within China’s physician credentialing framework retains “Clinical Medicine” as the overarching primary category for board certification. Notably, all experts included in this study were actively engaged in frontline emergency medicine clinical practice or residency training, ensuring that their expertise was directly relevant to the research topic.In terms of working experience, more than half of the experts (52.6%, *n* = 10) had over 15 years of clinical or teaching experience in emergency medicine, followed by those with 11–15 years of experience (31.6%, *n* = 6), and only 15.8% (*n* = 3) had 5–10 years of experience. All participating experts had more than 5 years of relevant experience, which guaranteed that they had sufficient practical and teaching experience in the emergency medicine field to provide authoritative consultation opinions.

Regarding educational background, the majority of the experts held a bachelor’s degree (68.4%, *n* = 13), while 21.1% (*n* = 4) had a master’s degree and 11.0% (*n* = 2) had a doctoral degree. All experts had an educational background of bachelor’s degree or above, which fully met the pre-set expert inclusion criteria.

All the participating experts were specialized in clinical emergency medicine, ensuring that the consultation results were closely aligned with the actual clinical needs and practical scenarios of emergency medicine. In terms of geographical distribution, most of the experts were from South China (84.0%, *n* = 16), and the remaining experts were distributed in North China, East China, and Northeast China, with 1 expert from each region (5.3% for each).

This regional distribution pattern can be partially attributed to the research team’s pre-existing collaborative network, which primarily encompasses well-developed tertiary hospitals in South China—where emergency medicine residency training systems are comparatively more mature, enabling their frontline experts to deliver more practical, experience-based insights into residency training requirements.

To mitigate potential regional bias in the expert panel, we proactively recruited additional experts from three other major regions of China (North China, East China, and Northeast China) to incorporate diverse cross-regional perspectives into the study. Furthermore, during the indicator screening phase, we explicitly excluded any indicators that experts identified as region-specific, to ensure the final indicator system reflected generalizable requirements for emergency residency training nationwide, rather than region-specific practices. Finally, the anonymous Delphi study design prevented dominant opinions from any single regional group from biasing the final expert consensus results.

**Table 2:** Demographic Characteristics of Experts (*n* = 19)

Category	Subcategory	Cases	Percentage (%)
Educational Background	Bachelor’s Degree	13	68.4
	Master’s Degree	4	21.1
	Doctoral Degree	2	11
Working Age	5–10	3	15.8
	11–15	6	31.6
	>15	10	52.6
Specialty Field	Clinical Medicine	19	100
Geographical Distribution	South China	16	84
	North China	1	5.3
	East China	1	5.3
	Northeast	1	5.3

### Expert response rates and authority coefficient

All 19 questionnaires were returned in both Delphi rounds. Both rounds achieved 100% valid response rates, confirming strong expert engagement [13]. The authority coefficient (Cr) was calculated as the mean of the judgment coefficient (Ca) and familiarity coefficient (Cs):

$$\:Cr=\left(Ca+Cs\right)/2$$

where *Cr* ≥ 0.70 is considered acceptable [14].

Specifically, the familiarity coefficient (Cs) was derived from the experts’ self-rating of their familiarity with each indicator, using a 5-point scale (1 = not familiar at all, 5 = extremely familiar).

The judgment coefficient (Ca) was based on the experts’ self-reported basis for their judgments, which included four categories: practical experience, theoretical analysis, reference to domestic literature, and reference to international literature. Each category was assigned a corresponding weight: practical experience (0.8), theoretical analysis (0.6), reference to domestic literature (0.5), and reference to international literature (0.4). Experts were asked to indicate which of these categories their judgment was based on, and the Ca score was calculated as the sum of the weights of the selected categories. This method is a standard approach for calculating expert authority coefficients in Delphi studies in medical education, which has been widely used in previous research to quantify the reliability of expert opinions.

Post-consultation, the study achieved *Cr* = 0.895, demonstrating strong expert authority.

### Concentration and coordination of expert opinions

The coefficient of variation (CV) and Kendall’s concordance coefficient (W) determined expert consensus divergence. Nineteen experts evaluated the importance of the 5 primary dimensions, 14 secondary indicators as well as 34 Core Competency Indicators.

The authority coefficient for both rounds of the Delphi survey was 0.895, which is above the threshold value of 0.7, confirming expert reliability. Expert consultation yielded CV ranges of 0-0.210 (Round 1) and 0-0.198 (Round 2). Kendall’s W values at each indicator level (second round of consultation) ranged from 0.566 to 0.634 (*p* < 0.005), indicating statistically significant agreement among experts with minimal opinion fluctuation. These results demonstrated high expert consensus validity (Table 3).

**Table 3:** Results of expert consultation coefficients across two Delphi rounds

Consultation Round	Evaluation Level	Coefficient of Variation (CV)	Kendall’s W	Chi-square Value	Degrees of Freedom	*P* -value
First round	First-level	0.046–0.133	0.2	15.209	4	< 0.05
	Second-level	0.064–0.151	0.078	19.382	13	> 0.05
	Third-level	0-0.210	0.08	53.111	35	< 0.05
Second round	First-level	0.046–0.11	0.319	24.267	4	< 0.05
	Second-level	0-0.196	0.613	151.492	13	< 0.05
	Third-level	0-0.198	0.566	354.739	33	< 0.05

### Training contents

Based on the results of the first round of consultation, our research group integrated the opinions of all experts, deleted the items with a coefficient of variation (CV) greater than 0.2, and revised the remaining items according to the experts’ suggestions. The specific operations are as follows:

- *Deleted items*: After discussion by the research group, we decided to delete the following items: − 5.1.2 “Actively seek to participate in the management of complex cases or difficult case discussions to improve own ability” − 5.2.2 “Have the internal driving force to step forward in the face of public health emergencies or disasters”.
- *Revision details*: - **Integration of third-level indicators**: Experts suggested adding a new third-level indicator: “Concept of loving injury, and having teaching awareness and ability”. However, the research group found that the content of this indicator overlapped with the existing indicator 4.3 “Empathy and Affinity”. Therefore, we integrated this content into the indicator 4.3, and the revised indicator 4.3.1 was finally determined as: “Can naturally express understanding and care for patients’ suffering, maintain humanistic care even under time pressure, and have the teaching ability of the concept of loving injury”.

In the first round of consultation, the Kendall’s W values ranged from 0.078 to 0.2, with only partial dimensions reaching statistical significance, indicating that expert opinions had not yet reached sufficient consensus at this stage. After revising the preliminary indicators based on the first-round feedback, we conducted the second-round consultation.

In the second round, the Kendall’s W values for all levels of indicators ranged from 0.319 to 0.613, all of which were statistically significant (*P* < 0.05). Meanwhile, the range of CV decreased to 0-0.198, with all indicators meeting the pre-set retention threshold, indicating that the consistency of expert opinions was significantly improved compared to the first round, demonstrating good inter-rater reliability. No further modifications were suggested by the experts in this round.

Following this, the research team held discussions based on the feedback from both Delphi rounds and finalized the indicator system. This resulted in 5 first-level indicators, 14 s-level indicators, and 34 third-level indicators.

These indicators are organized into a standardized hierarchical three-tier system:

1. First-level indicators: These are the broad, top-level dimensions derived from the iceberg competency model, representing the core competency categories: motivation, trait, self-concept, knowledge, and skill. These dimensions define the overall framework of the competency system, drawing a clear distinction between explicit surface-level competencies and implicit deep-level competencies.
2. Second-level indicators: These are the sub-dimensions nested under each first-level dimension, which break down the broad top-level categories into more specific, actionable competency domains. For instance, under the “skill” first-level dimension, the corresponding second-level indicators include clinical procedural skills, clinical decision-making skills, and interpersonal communication and coordination skills.
3. Third-level indicators: These are the most granular, behaviorally anchored indicators nested within each second-level domain. These concrete, assessable items are directly applicable to the evaluation of resident competencies, with a typical example being “proficiency in cardiopulmonary resuscitation.” This hierarchical structure enables the indicator system to support both global competency assessment and detailed, item-specific evaluation of residents’ training progress.

Given the large volume of detailed indicator content, we placed the complete evaluation indicator scheme in the supplementary material (Additional file 1: Table S1), and the other core results are presented in Tables 4 and 5.

**Table 4:** First-level indicator layer second round expert consultation results

Level 1	Standard deviation	Average	Coefficient of variation	weight
Knowledge	0.452	4.263	0.106	0.065
Skills	0.229	4.947	0.046	0.405
Self-Concept	0.496	4.632	0.107	0.163
Traits	0.419	4.790	0.088	0.268
Motivation	0.507	4.421	0.115	0.100

**Table 5:** Results of the second round of expert consultation for the second-level indicators

Level 1	Level 2	Standard deviation	Average	Coefficient of variation	weight
1 Knowledge	1.1 Medical Foundations	0.315	4.895	0.064	0.358
	1.2 Clinical Expertise	0.000	5.000	0.000	0.568
	1.3 Humanities & Social Sciences	0.658	3.895	0.169	0.075
2 Skills	2.1 Clinical Procedures	0.315	4.895	0.064	0.311
	2.2 Clinical Reasoning	0.229	4.947	0.046	0.493
	2.3 Communication & Teamwork	0.375	4.842	0.077	0.196
3 Self-Concept	3.1 Role Identity	0.671	3.684	0.182	0.131
	3.2 Professional Values	0.452	3.737	0.121	0.208
	3.3 Self-Regulation	0.507	4.421	0.115	0.661
4. Traits	4.1 Resilience	0.653	3.737	0.175	0.143
	4.2 Precision & Attention	0.734	3.737	0.196	0.143
	4.3 Empathy & Collaboration	0.582	4.684	0.124	0.714
5. Motivation	5.1 Achievement & Dedication.	0.315	4.895	0.064	0.857
	5.2Innovation & Integration	0.582	3.684	0.158	0.143

## Discussion

### Interpretation and structural analysis of the core competency indicator system

In this study, we developed a core competency indicator system for emergency residency training through the Delphi method, which finally included 5 first-level indicators (motivation, trait, self-concept, knowledge, and skill), 14 s-level indicators, and 34 third-level indicators. To our knowledge, this is the first study that fully introduced Spencer’s Iceberg Competency Model into the construction of emergency residency training indicators in China, filling the gap of traditional research that only focused on explicit clinical abilities.

Weight analysis of the first-level indicators showed that: skill had the highest single weight at 0.405, followed by trait at 0.268, self-concept at 0.163, motivation at 0.100, and knowledge at the lowest weight of 0.065. Specifically, the explicit foundational competencies (knowledge and skill) that lie above the iceberg accounted for 47% of the total weight, while the implicit differentiating competencies (self-concept, trait, and motivation) that lie below the iceberg accounted for 53% of the total weight. This finding is particularly noteworthy: it indicates that emergency medicine experts generally agree that the implicit deep-level competencies—those often overlooked in conventional residency training—are equally critical for emergency physicians as the explicit foundational knowledge and skills. Although the implicit competencies carry a marginally higher overall weight, this finding highlights that these often-overlooked competencies are not secondary to clinical skills and knowledge, but rather represent core components of competent emergency practice, thus deserving equal emphasis in residency training curricula.

These implicit traits are the core factor that determines physicians’ behavioral patterns and long-term development potential in high-pressure emergency environments [15]. The weight distribution highlights a novel quality-oriented assessment concept, rather than the traditional knowledge-skill oriented mode: skill still occupied the highest single weight, reflecting that clinical skill remains the primary threshold for emergency residents to take up their posts. The weight of the skill dimension was 0.405, far higher than the other four dimensions, fully indicating that for emergency residents, clinical skill is the most core entry requirement. This structure fully aligns with the hierarchical logic of Spencer’s classic competency model, which progresses from explicit surface traits to implicit deep-seated characteristics [16].

The constructed system is characterized by its close alignment with the practical context of emergency medicine [17]: the emergency department is the frontline of acute and critical care in hospitals, with the core feature of “time is life”. Emergency physicians face patients whose lives may be endangered at any time, and the core requirement is rapid disposal: clinical operation skills such as cardiopulmonary resuscitation, endotracheal intubation, and trauma hemostasis, as well as non-operative skills such as rapid priority judgment, are the key factors that directly determine the prognosis of emergency patients [18]. Even with strong professional motivation, without proficient clinical skills, physicians cannot deliver life-saving interventions during the golden time window of emergency care, which is the biggest difference between emergency medicine and other departments.

The results of this study also match the core training goal of residency training: the essence of residency training is to train “theoretical students” from medical schools into “clinical physicians” who can work independently. Medical students have already mastered sufficient basic medical knowledge during their college years, and the core shortcoming in the residency stage is the clinical skill to translate theoretical knowledge into practice. The high weight of 0.268 for the trait dimension fully indicates that experts have reached a consensus: in the high-intensity and high-risk emergency environment, personal traits such as stress resistance and emotional stability are far more important than the basic medical knowledge that residents have already mastered during their college education. Furthermore, these implicit traits and motivation are long-term professional literacy, which cannot be rapidly acquired by residents during the 2–3 years of residency training, nor are they the core entry threshold for the residency stage. Within the trait dimension, “empathy and affinity” was highly valued by experts, which is the key soft skill to manage the prevalent high-anxiety atmosphere in emergency departments and build harmonious physician-patient relationships [19]. The motivation dimension emphasized the translation of evidence-based medicine into concrete behaviors, such as “adjusting treatment plans based on the best available evidence”, which aligns with the transformation trend of modern emergency medicine [20].

### Comparison with existing competency frameworks and standards

The competency framework developed in this study demonstrates conceptual alignment with core elements of the ACGME’s six core competencies [14], and China’s residency training standards [21], particularly in emphasizing medical knowledge, patient care, and interpersonal/communication skills. Such concordance across international and domestic benchmarks validates that the proposed framework captures universally recognized priorities in clinical physician development.

The unique value of our system lies in its deeper responsiveness to the specific context of emergency medicine and its complementary enhancement of model completeness, which is primarily manifested in three aspects:

1. It systematically incorporates and delineates implicit qualities. Existing frameworks (e.g., the “Professionalism” domain in ACGME) often provide more detailed descriptions of explicit competencies while addressing implicit psychological traits in a relatively broad manner. This study explicitly designates motivation, trait, and self-concept as first-level indicators. Specifically, it delineates traits such as “stress resilience” and “empathy and affinity,” directly addressing the specific psychological competency demands placed on physicians in high-pressure emergency environments, thereby supplementing the current standards in this under-explored area.
2. It achieves a precise, context-specific refinement of competencies for emergency scenarios. Examples include the knowledge dimension’s “subspecialty knowledge in disaster medicine and toxicology” (third-level weight 0.297) and the skill dimension’s “Advanced Cardiac Life Support (ACLS)” (0.540) and “risk prioritization under time pressure” (0.559). These are unique “emergency-specific” competencies that are often subsumed under generalized categories like “medical knowledge” or “patient care” in existing frameworks, without highlighting their contextual specificity.
3. The motivation dimension’s “evidence-based knowledge and practical ability” (second-level weight 0.857) refines the broad requirement of “evidence-based medicine” from a general “practice based on learning” into the concrete behavior of “adjusting treatment plans based on the best available evidence.” This refinement more closely aligns with the transformative trend toward “evidence-based orientation” in modern emergency medicine.

### The core theoretical value of the constructed indicator system

The core theoretical value of this study lies in verifying the applicability of the competency model in the field of emergency residency training, and transforming the abstract competency theory into specific, assessable behavioral indicators. Spencer’s classic model emphasizes the hierarchical logic of “implicit deep-seated traits - explicit clinical abilities”, but previous studies mostly stayed at the level of general competencies, without refinement combined with the context of specific specialties. By introducing emergency-specific indicators such as “stress resilience” and “evidence-based practice”, this study transformed the model from a “general framework” into an “exclusive tool for emergency residency training”. For instance, stress resilience is not a core competency for all specialties, but it is a differentiating competency for emergency physicians to cope with the “high-pressure, high-uncertainty” emergency scenarios. This transformation enriches the research on the competency model in the field of specialized residency training.

### Methodological advantages and limitations

The methodology of this study has several strengths: first, we achieved a 100% response rate in the two rounds of Delphi consultation, and the expert authority coefficient was 0.895, which was far higher than the threshold of 0.7, indicating that the included experts had high enthusiasm and authority, ensuring the reliability of the results. In addition, we fully introduced the iceberg competency model into the indicator construction, which made up for the deficiency of traditional studies that only focused on explicit knowledge and skill.

Nevertheless, there are still some limitations that need to be improved in future studies:

First, the Delphi method itself has inherent limitations. Although the anonymous design avoided the influence of group pressure, the results are still based on the subjective judgments of experts, which may be affected by experts’ personal experience and professional preferences.

Second, the expert sample still had a relatively higher proportion of participants from South China (84%). This can be attributed to the highly stringent expert inclusion criteria we adopted: we only recruited senior experts with at least 5 years of specialized experience in emergency medicine residency training, to ensure the reliability of the consultation results. As an independent specialty, emergency medicine underwent earlier development in South China, and the standardized residency training system for emergency medicine has been in place for a longer duration in this region. Consequently, this region has a larger pool of experts who meet our strict inclusion criteria, resulting in the higher proportion of participants from South China.

We conducted an additional consistency analysis of expert ratings across regions, which revealed no statistically significant differences in the importance scores of all indicators between experts from South China and those from other regions. This further confirms that the final indicator system is generalizable across different regions of China, rather than being limited to the South China context. Furthermore, tertiary emergency departments in South China have accumulated rich experience in managing high patient volumes, high workload pressure, and complex clinical scenarios—this represents the future development trajectory of emergency departments in other regions of China. Thus, the practical insights from these experts can also provide forward-looking reference for residency training nationwide.

The scenario characteristics of primary emergency departments are significantly different from those of tertiary hospitals, and their demand for core competencies may focus more on “management of common emergencies in primary care” and “coordination of upper and lower level referral” [22]. In addition, all the experts included in this study were from China, which means the results are entirely based on China’s medical context, which may limit the generalizability of our findings to other countries with different medical systems.

Third, the empirical validity of the indicator system remains to be verified. This study constructed a “consensus-based framework”, and its predictive validity (the correlation with clinical performance and patient outcomes) has not been empirically tested. Although the Delphi method ensured the content validity (experts recognized the relevance of the indicators), whether the indicators can distinguish “competent/incompetent residents” and predict clinical outcomes such as “rescue success rate and medical dispute rate” still needs to be verified in future multi-center studies to confirm its practical application value.

### Future study

The framework constructed in this study is a consensus-based framework, and its predictive validity (that is, whether the indicator scores can predict clinical performance and patient outcomes) has not been verified yet. Future studies need to conduct longitudinal cohort studies and multi-center empirical tests to address this:

- Correlate with clinical outcomes: Track the achievement degree of the indicators of residents at different training stages, and analyze its correlation with objective clinical indicators (such as emergency rescue success rate, medical dispute incidence, and patient satisfaction) and competency assessment results (such as the passing rate of graduation assessment and specialist physician certification).
- Develop supporting assessment tools: Transform the third-level indicators into specific assessment tools, and test the reliability and validity of these tools, to solve the practical pain point that “we have the indicators but do not know how to conduct quantitative assessment“ [23].

## Conclusion

Through two rounds of Delphi expert consultation, this study constructed a core competency indicator system for emergency medicine residency training—covering 5 first-level indicators (Motivation, Traits, Self-Concept, Knowledge, and Skills), 14 s-level indicators, and 34 third-level indicators—that systematically addressed the unique scenario characteristics of emergency medicine, including high work pressure, high clinical uncertainty, time urgency, and prominent humanistic care needs, and provided a theoretical framework and practical tool for the standardized management of emergency medicine residency training.

## Supplementary Information

Supplementary Material 1.

## Data Availability

The data that support the findings of this study are available from the corresponding author upon reasonable request.
