# Supplementary material for: Development of core competency indicators for emergency medicine residents based on the competency model: a Delphi and analytic hierarchy process study
Source: BMC Med Educ. 2026 Jul 7;26:1549. doi: 10.1186/s12909-026-09876-3 (PMC13625334; doi:10.1186/s12909-026-09876-3)
Supplement: Supplementary file 1 [file 12909_2026_9876_MOESM1_ESM.docx]

Table S1. Results of the Second Round of Expert Consultation for the Third-Level Indicators

| Level 1 | Level 2 | Level 3 | Standard deviation | Average | Coefficient of variation | weight |
| --- | --- | --- | --- | --- | --- | --- |
| 1.Knowledge | 1.1 Medical Foundations | 1.1.1 Ability to apply pathophysiological knowledge of common acute and critical illnesses to clinical practice. | 0.631 | 4.211 | 0.150 | 0.218 |
|  |  | 1.1.2 Ability to safely apply emergency medication pharmacology in clinical scenarios. | 0.315 | 4.895 | 0.064 | 0.691 |
|  |  | 1.1.3 Ability to integrate etiological and epidemiological knowledge into the management of infectious and endocrine emergencies. | 0.375 | 3.842 | 0.098 | 0.091 |
|  | 1.2 Clinical Expertise | 1.2.1 Ability to proficiently conduct diagnosis and differential diagnosis for common system-based emergency conditions. | 0.496 | 4.632 | 0.107 | 0.163 |
|  |  | 1.2.2 Ability to implement core international emergency protocols (e.g., ACLS, ATLS) and emergency green channel procedures. | 0.000 | 5.000 | 0.000 | 0.540 |
|  |  | 1.2.3 Ability to apply subspecialty knowledge in disaster medicine, toxicology, and emergency ultrasound to emergency care. | 0.419 | 4.790 | 0.088 | 0.297 |
|  | 1.3 Humanities & Social Sciences | 1.3.1 Ability to adhere to medical ethics and healthcare regulations in emergency practice. | 0.375 | 4.842 | 0.077 | 0.875 |
|  |  | 1.3.2 Ability to apply psychodynamic and cultural competency principles in doctor-patient communication. | 0.507 | 3.421 | 0.148 | 0.125 |
| 2. Skills | 2.1 Clinical Procedures | 2.1.1 Ability to proficiently perform basic emergency clinical procedures (e.g., venous/arterial puncture, catheterization, thoracentesis, lumbar puncture). | 0.229 | 4.947 | 0.046 | 0.250 |
|  |  | 2.1.2 Ability to independently or collaboratively deliver advanced life support interventions (e.g., intubation, mechanical ventilation, defibrillation). | 0.000 | 5.000 | 0.000 | 0.500 |
|  |  | 2.1.3 Ability to competently perform rapid bedside ultrasound assessment. | 0.229 | 4.947 | 0.046 | 0.250 |
|  | 2.2 Clinical Reasoning | 2.2.1 Ability to conduct rapid history-taking, focused physical examination, and identify critical warning signs. | 0.452 | 4.737 | 0.096 | 0.352 |
|  |  | 2.2.2 Ability to generate prioritized differential diagnoses and evidence-based diagnostic plans. | 0.780 | 3.947 | 0.198 | 0.089 |
|  |  | 2.2.3 Ability to conduct risk assessment and dynamically adjust treatment plans under time pressure. | 0.375 | 4.842 | 0.077 | 0.559 |
|  | 2.3Communication & Teamwork | 2.3.1 Ability to conduct structured handover and effective team communication. | 0.315 | 4.895 | 0.064 | 0.348 |
|  |  | 2.3.2 Ability to communicate prognosis, informed consent, and bad news to patients and families. | 0.671 | 3.684 | 0.182 | 0.070 |
|  |  | 2.3.3 Ability to effectively lead or participate in resuscitation teams under high-pressure scenarios. | 0.229 | 4.947 | 0.046 | 0.582 |
| 3. Self-Concept | 3.1 Role Identity | 3.1.1 Ability to internalize the emergency physician role as a life-saver and team leader in clinical practice. | 0.780 | 4.053 | 0.192 | 0.167 |
|  |  | 3.1.2 Ability to maintain calmness and confidence when facing high-pressure clinical scenarios. | 0.315 | 4.895 | 0.064 | 0.833 |
|  | 3.2 Professional Values | 3.2.1 Ability to prioritize patient safety and proactively mitigate clinical risks. | 0.507 | 4.579 | 0.111 | 0.140 |
|  |  | 3.2.2 Ability to maintain commitment to lifelong learning and continuous professional improvement. | 0.375 | 4.842 | 0.077 | 0.333 |
|  |  | 3.2.3 Ability to recognize personal expertise limits and proactively seek consultations when needed. | 0.229 | 4.947 | 0.046 | 0.528 |
|  | 3.3 Self-Regulation | 3.3.1 Ability to conduct reflective practice and achieve emotional recovery after clinical setbacks. | 0.315 | 4.105 | 0.077 | 0.167 |
|  |  | 3.3.2 Ability to identify personal knowledge gaps and develop proactive learning plans. | 0.229 | 4.947 | 0.046 | 0.833 |
| 4. Traits | 4.1 Resilience | 4.1.1 Ability to maintain emotional stability and clear thinking under prolonged high-stress conditions. | 0.229 | 4.947 | 0.046 | 0.833 |
|  |  | 4.1.2 Ability to maintain patience and adapt to uncertainty or incomplete clinical information. | 0.602 | 4.158 | 0.145 | 0.167 |
|  | 4.2 Precision & Attention | 4.2.1 Ability to strictly adhere to the "three checks, seven verifications" protocol during complex emergency procedures. | 0.315 | 4.895 | 0.064 | 0.750 |
|  |  | 4.2.2 Ability to produce clear, logical clinical documentation that accurately reflects clinical reasoning. | 0.513 | 4.526 | 0.113 | 0.250 |
|  | 4.3 Empathy & Collaboration | 4.3.1 Ability to provide compassionate care for patients' suffering, maintain humanistic care even under time constraints, and deliver teaching on the concept of loving injury. | 0.684 | 3.632 | 0.188 | 0.125 |
|  |  | 4.3.2 Ability to build trust and collaborate with diverse interdisciplinary team members. | 0.315 | 4.895 | 0.064 | 0.875 |
| 5.Motivation | 5.1Achievement & Dedication | 5.1.1 Ability to set self-imposed professional standards that exceed basic clinical competency requirements. | 0.229 | 4.947 | 0.046 | 0.833 |
|  |  | 5.1.2 Ability to recognize and internalize the societal value of emergency medicine practice. | 0.524 | 3.947 | 0.133 | 0.167 |
|  | 5.2 Innovation & Integration | 5.2.1 Ability to conduct evidence-based practice and translate research evidence into clinical emergency care. | 0.229 | 4.947 | 0.046 | 0.833 |
|  |  | 5.2.2 Ability to conduct clinical research to improve emergency care practice. | 0.705 | 4.053 | 0.174 | 0.167 |
